# Stuttering as a signal of encephalopathy associated with toripalimab in a pancreatic ductal adenocarcinoma patient: a case report

**DOI:** 10.1186/s12883-023-03140-7

**Published:** 2023-03-04

**Authors:** Jianping He, Xi Chen, Ke Cheng, Wanrui Lv, Dan Cao, Zhiping Li

**Affiliations:** 1 Abdominal Oncology Ward, West China Hospital, Sichuan University, Cancer Center, Chengdu, China; 2 Division of Radiotherapy, West China Hospital, Sichuan University, Cancer Center, Chengdu, China

**Keywords:** Encephalopathy, Immune checkpoint inhibitor, Immune-related adverse event, Pancreatic ductal adenocarcinoma, Toripalimab

## Abstract

**Background:**

Immune checkpoint inhibitor (ICI) combined with chemotherapy has exhibited promising results in small sample studies of pancreatic cancer patients. The efficacy of toripalimab, a programmed cell death protein 1 (PD-1) monoclonal antibody has been explored in the previous studies and it was established that immune-related adverse events (irAEs) associated with administration of this drug deserve proper attention and adequate management.

**Case presentation:**

A 43-year-old female patient with advanced pancreatic ductal adenocarcinoma (PDAC) was treated with toripalimab in combination with gemcitabine and nab-paclitaxel (T-GA) as the first-line treatment. She developed immune-related encephalopathy with stuttering as the main clinical symptom and Magnetic resonance imaging (MRI) showed multiple cerebral white matter demyelination changes, concomitant with asymptomatic cardiac enzyme elevation and hypothyroidism. The symptoms resolved after the discontinuation of toripalimab and corticosteroid treatment.

**Conclusions:**

Stuttering might be an early sign of neurotoxicity which can be easily neglected during the treatment. These findings provide guidance for the identification of these rare and occult neurological irAEs (n-irAEs) in the clinical practice.

## Background

The advent of various immune checkpoint inhibitors (ICIs) have revolutionized the treatment of solid tumors and hematological malignancies in recent years [1]. The application of ICIs in pancreatic ductal adenocarcinoma (PDAC), which is one of the most aggressive solid tumors has also received significant attention. Currently, programmed cell death protein 1/ligand 1 (PD-1/L1) monotherapy has exhibited a limited response in PDAC patients [2, 3]; however, there are growing evidences to indicate that the combination of humanized IgG monoclonal antibodies and chemotherapeutic agents can display durable responses in clinical trials at the various stages of treatment in PDAC patients [4, 5].

Toripalimab, a humanized monoclonal anti-PD-1 antibody, has been approved by FDA for the treatment of advanced melanoma [6], nasopharyngeal carcinoma [7], urothelial carcinoma [8] and esophageal squamous cell carcinoma [9]. We are currently conducting a prospective open-label, phase Ib/II clinical study to evaluate the potential efficacy and safety of the combination of toripalimab, gemcitabine and nab-paclitaxel (T-GA) as a first-line treatment for unresectable PDAC [10]. Interestingly, it has been reported that T-GA triple combination exhibited a favorable response and manageable toxicity in advanced PDAC patients in preliminary results [11]. However, it is worth mentioning that administration of ICIs has been found to be closely associated with a wide range of immune-mediated toxicities, called immune-related adverse events (irAEs), that can affect almost every organ system [12]. Here, we report the case of toripalimab-induced multisystem irAEs of encephalopathy, cardiotoxicity, and thyroid dysfunction as a reminder of the potential risks associated with application of these novel immunotherapeutic agents.

## Case presentation

**Fig. 1:**
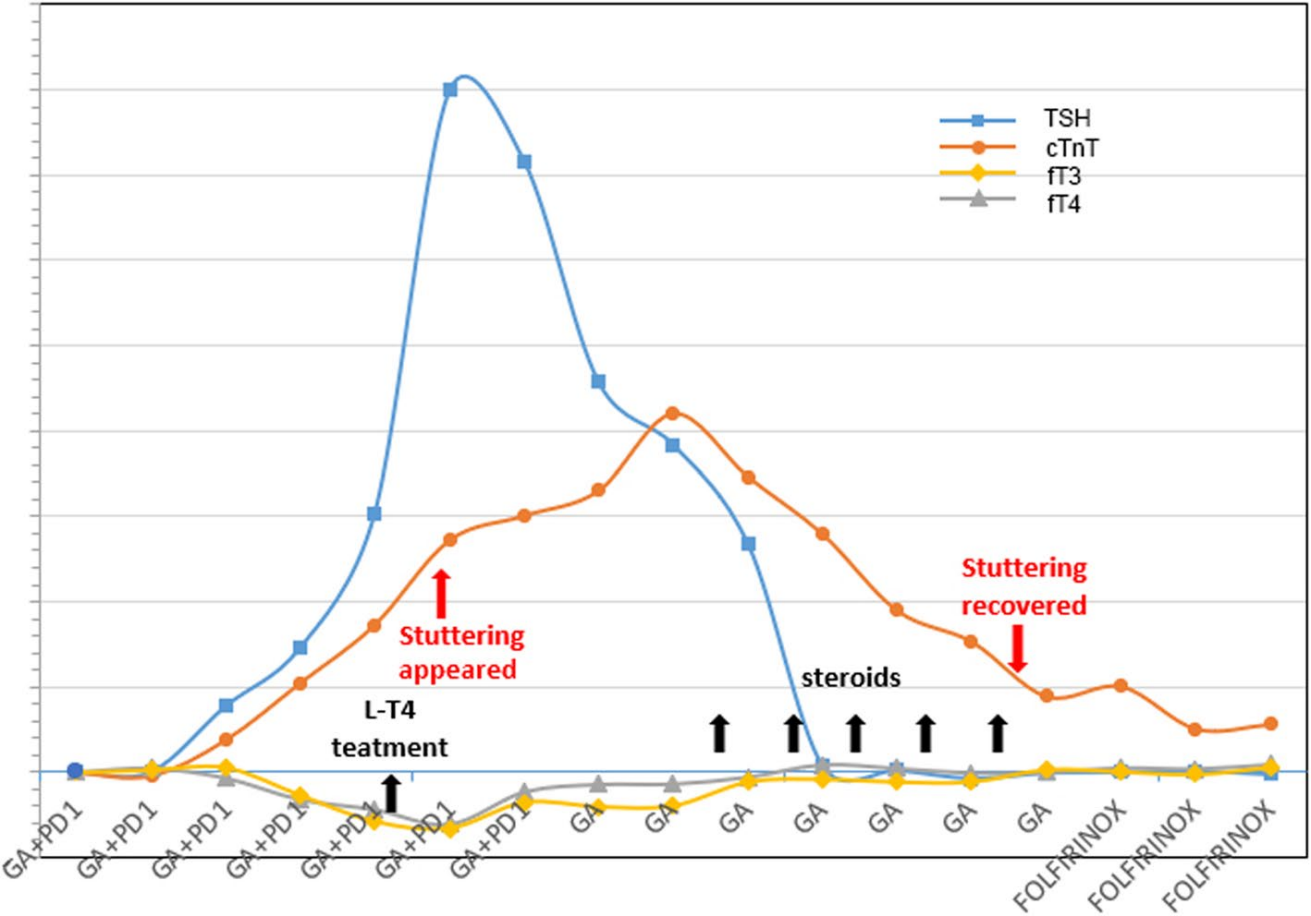
A schematic diagram of observed changes in stuttering, myocardial markers, and thyroid function. Upon the administration of immunochemotherapy, troponin and TSH gradually increased, fT3 and fT4 steadily decreased, and stuttering occurred. After the discontinuation of immunotherapy and initiation of glucocorticoid treatment, all indicators returned to the normal levels. cTnT: cardiac troponins T; TSH: thyrotropin; fT3: free triiodothyronine; fT4: free thyroxine

A 43-year-old woman was admitted to our hospital due to abdominal pain, and she was suspected of having a pancreatic tumor after imaging examinations. She was subjected to radical surgery and was pathologically diagnosed with PDAC. In immunohistochemistry investigations, EMA, CK19, CK8/18, CA19-9, and MIB-1 (20%) were all positive, while CA125 and DPC-4 were found to be negative. However, shortly after the surgery, new metastatic lesions in the liver and peripancreatic lymph nodes appeared, and she began receiving T-GA (gemcitabine 1000 mg/m^2^ and nab-paclitaxel 125 mg/m^2^ on day 1 and day 8 and toripalimab 240 mg on day 1, administered every 3 weeks) as the first-line therapy in May 2019, despite the fact that genetic analysis of the tumor tissues indicated a low tumor mutation burden, microsatellite stability, and negative PD-L1 expression. After completion of 4 cycles of the treatment, the levels of asymptomatic myocardial markers were increased, but the thyroid function was found to be substantially decreased (Fig. 1). After daily oral administration of 75 µg levothyroxine, the thyroid function index improved significantly.

**Fig. 2:**
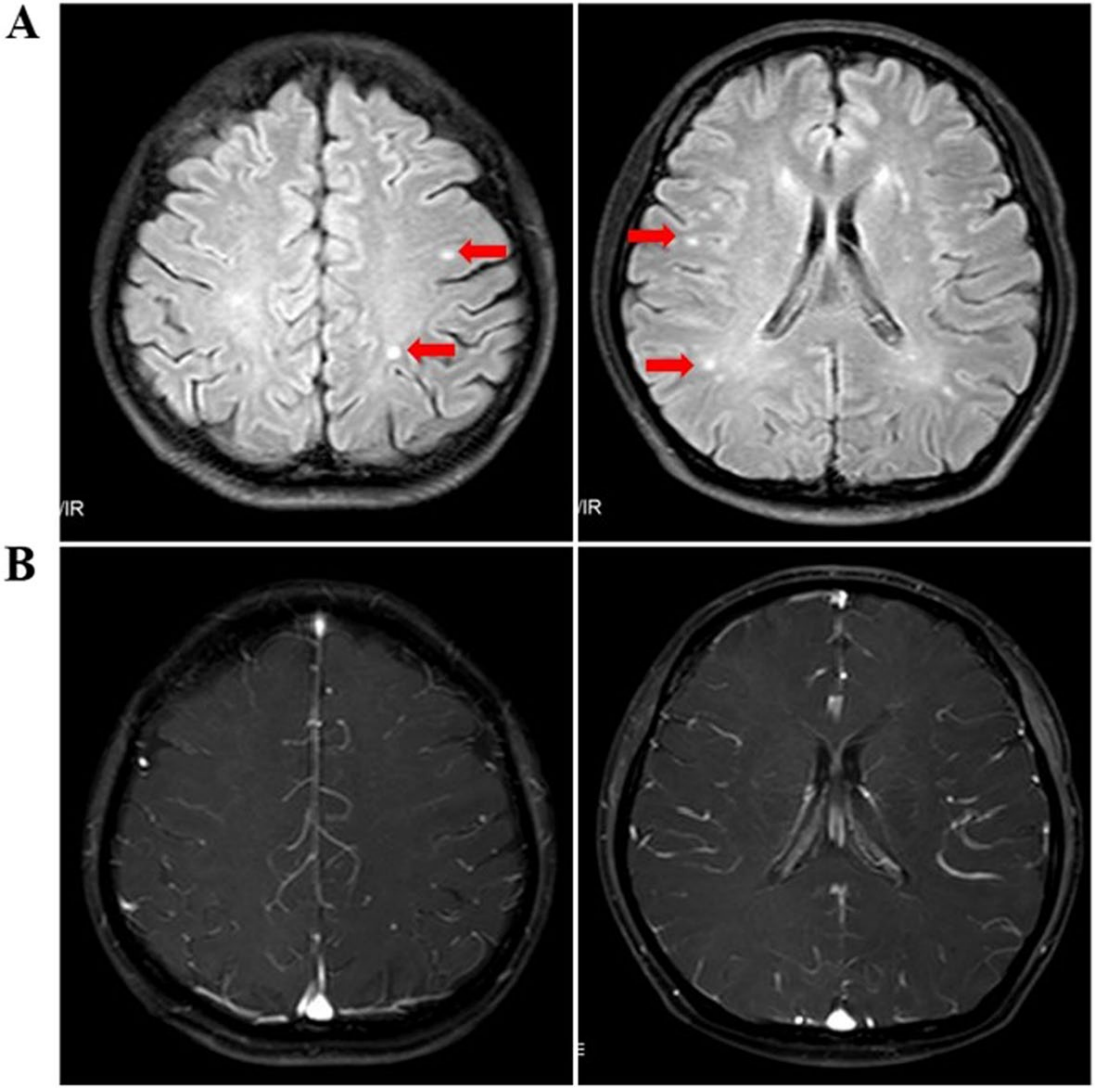
MRI performance of the patient. **A** T2 Flair hyperintense signal in the multiple areas of the cerebral white matter, primarily in the parietal lobes but also involving the posterior frontal lobes, corpus callosum, and right brachium pontis were observed. **B** None of these lesions were enhanced following the contrast administration. In addition, no restricted diffusion was present and no significant mass effect or midline shift was identified

Notably, two weeks after administration of the fifth dose of T-GA, the patient experienced speech impairment, stuttering, with frequent halts, airflow blocks and repetition during this period. As a professional woman, she immediately detected the symptoms associated with imperceptible stuttering during the treatment because she had been articulate for the previous several decades. In addition, the non-fluent speech was persistent which aggravated gradually and was accompanied by slow thinking, self-consciously “stupid, dull”, without fever, headache, vomiting, fatigue, stiff neck or other psychiatric symptoms. A panel of serological antibodies (ANNA1, PCA-1, ANNA2, anti-CV2, anti-amphiphysin, anti-Ma1, anti-Ma2, anti-SOX1, DNER, anti-Zic4, anti-GAD65, anti-titin, anti-recoverin, and anti-PKCγ) that have been implicated in the neurological paraneoplastic syndromes was found to be negative. Magnetic resonance imaging (MRI) revealed the presence of the cerebral white matter demyelinating polyneuropathy but no evidence of encephalitis or cancer metastasis was found (Fig. 2). A nerve conduction study, cerebrospinal fluid test and electroencephalogram were not conducted due to the refusal by the patient. Moreover, the levels of elevated myocardial markers peaked at grade 3 cardiotoxicity according to CTCAE 4.0. We considered that stuttering could be possibly attributable to encephalopathy, and toripalimab as the possible underlying cause. Hence, it was immediately discontinued and only chemotherapy was maintained. Indeed, encephalopathy and cardiotoxicity rapidly improved after the discontinuation of toripalimab treatment and administering intravenous corticosteroids. As the patient’s stuttering completely recovered, comprehension as well as thought reaction speed was also found to be improved. The various laboratory indicators also showed marked improvement and hence the doses of steroids were gradually tapered and stopped. Toripalimab was permanently discontinued in the light of the observed neurological and cardiac toxicity, as a rare emerging toxicity that may lead to a fatal outcome.

**Fig. 3:**
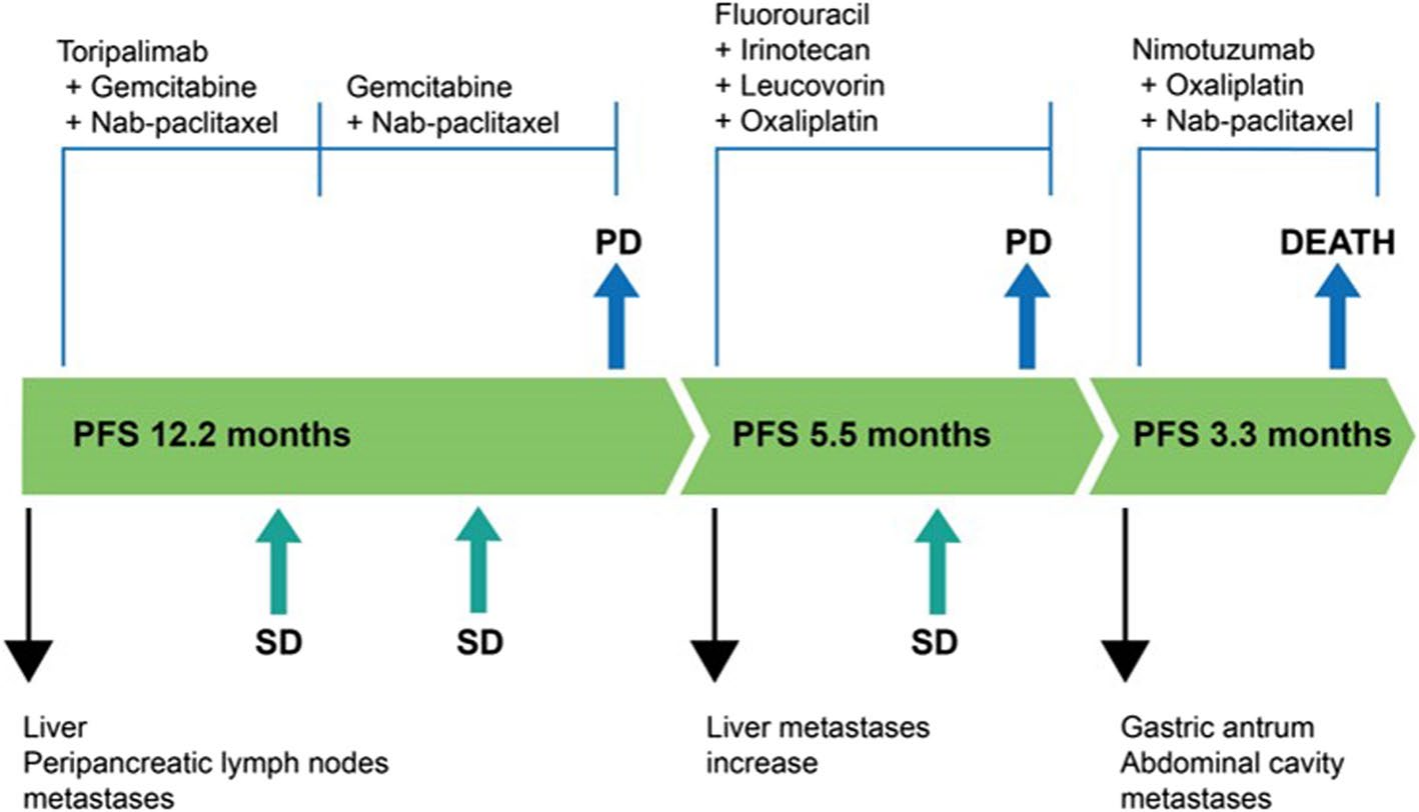
Summary of the disease course and the treatment process. The patient was subjected to third-line therapy and OS was found to be 21 months. PD: Progressive Disease; PFS: Progression-free survival; OS: Overall survival

During the follow-up treatment, the patient was treated with the second-line regimen of fluorouracil, irinotecan, leucovorin, oxaliplatin (FOLFIRINOX) due to the increase in the liver metastases. However, later once the multiple metastases appeared in the gastric antrum and abdominal cavity, the patient was treated with nimotuzumab in combination with oxaliplatin and nab-paclitaxel based on the results of mini patient-derived xenograft drug sensitivity test, which has the lowest relative proliferative power of tumor cells, but died after 3.3 months of progression-free survival (PFS). Overall, from the start of first-line treatment to death, the overall survival (OS) of the patient was 21 months (Fig. 3). The patient did not experience any recurrence of the neurological, cardiac, or thyroid toxicity throughout the subsequent follow-up period.

## Discussion and conclusions

It has been reported that neurological irAEs (n-irAEs) can occur in approximately 1% of patients receiving ICIs as monotherapy and in 2-3% of patients receiving it as a combination therapy [13]. The most common n-irAEs include myasthenia gravis, encephalitis/meningitis, inflammatory polyneuropathy (e.g., Green-Barre syndrome), and peripheral neuropathies [14, 15]. Although neurotoxicity is a rare occurrence, it can be serious and even prove to be fatal. It has been reported that among the 3545 patients treated with ICIs, the rate of death due to irAEs was 0.6%, among which 43% were cardiac and neurological events. Analysis of the WHO Drug Alert database for fatal irAEs has revealed that anti-PD-1/PD-L1-related neurological events accounted for approximately 15% of deaths, second only to pneumonia (35%) and hepatitis (22%) [16]. Moreover, the diagnosis of n-irAEs relatively complex and currently under recognized, thereby exacerbating potential risks associated with them.

Here, we report a female patient with advanced PDAC who presented after 5 cycles of treatment with T-GA with symptoms of stuttering as well as slow thinking as the main clinical manifestation. The neurotoxicity of GA chemotherapy has been reported primarily in the periphery [17]. Although there are few studies on pancreatic cancer immunotherapy, according to two prior published studies, pembrolizumab or nivolumab combined with gemcitabine plus nab-papaclitaxel displayed no central neurotoxicity [18, 19], so the likelihood that the interaction between ICIs and chemotherapy drugs will induce encephalopathy or encephalitis is relatively low and hence more investigations as well as studies will be required to prove the potential link. In contrast, the central neurotoxicity caused by ICIs has drawn significant attention, particularly in cases of lung cancer and melanoma patients [20, 21], who are frequently administered ICIs. There is, however, little information on whether ICIs can cause central neurotoxicity in the pancreatic cancer patients. It is plausible to believe that the toxicity is mainly driven by ICI based on the possible association between the time of drug usage and the improvement in neurotoxicity following the discontinuation of toripalimab. Therefore, she was diagnosed with toripalimab-related n-irAE accompanied with multiple demyelinating imaging changes, in combination with asymptomatic myocarditis and hypothyroidism. This n-irAE displayed a different timing of onset as well as the symptoms and we found that intracranial imaging changed markedly compared to the case of early-onset cognitive dysfunction along with overt signs and symptoms of cerebellar involvement as reported earlier by Zhou et al. [22]. In an analysis of 347 patients treated with pembrolizumab or nivolumab, the median time to onset of n-irAEs was 5.5 cycles. In addition, approximately half of the patients experienced other major systemic immune-mediated complications, including hypothyroidism, colitis, and hepatitis [20]. Stuttering has been reported as the first sign of CAR-T-cell-associated encephalopathy in women affected with lymphoma. Thus, given the subsequent rapid neurological deterioration observed in this patient, speech disfluency appears to be an early sign of neurotoxicity [23]. However, we have not found any prior reports related to n-irAEs induced by ICIs with stuttering as the main clinical manifestation. Stuttering as a mild symptom may serve as an early signal of n-irAEs. Once the early diagnosis of n-irAEs is established, it can be easily identified and intervened early to prevent the occurrence of severe AEs.

The lack of specificity in the diagnosis of n-irAEs often requires recourse to exclusionary diagnostic thinking, but in this case antibodies elicited in response to paraneoplastic neurological syndrome were not detected. However, subsequent MRI imaging showed a T2 Flair hyperintense signal in the multiple areas of the cerebral white matter, primarily in the parietal lobes but also affecting the posterior frontal lobes, corpus callosum, and right brachium pontis. In addition, none of these lesions were observed to be enhanced following the contrast administration and no restricted diffusion was present. Moreover, no significant mass effect or midline shift was identified. These findings were indicative of acute demyelinating encephalomyelitis, and she was thought to be suffering from immunotherapy-induced demyelination. Among the patients in whom MRI changes can be observed for ICI-induced neurotoxicity, manifestation of the central demyelination abnormalities has been reported to be the most common [24]. It can also present itself as the diffusion limitation of the limbic system [25], large lesions with mild enhancement [26] or focal T2 high signal [27]. For pancreatic cancer, a tumor with a relatively low risk of brain metastases, the current guidelines do not recommend routine head MRI procedure as a baseline screening prior to the initiation of the treatment. Therefore, no head MRI was conducted in imitation of this patient before starting the treatment. However, if routine MRI could be performed on all pre-ICI patients, pre- and post-treatment imaging comparisons could be extremely helpful in confirming and identifying the various treatment-related AEs. However, for such rare n-irAEs, it can lead to excessive health economic waste and this aspect should be taken into consideration as well.

Considering the possible further progression of the patient's n-irAEs and the combined increase in the level of cardiac enzymes in grade 3, it was reasonable to discontinue toripalimab. However, due to the high malignancy of pancreatic cancer, chemotherapy was not suspended completely. Current treatment guidelines usually recommend starting corticosteroids therapy as soon as the diagnosis of n-irAEs is confirmed. In general, oral corticosteroids are used for the management of the mild to moderate disease, whereas intravenous methylprednisolone infusions are mainly used for the more severe symptoms. Intravenous immunoglobulin or plasma exchange are employed for more severe diseases or for patients who are refractory to the steroids alone. Antibody-positive autoimmune encephalopathy can also be treated with rituximab to induce massive T-cell depletion [13, 28]. Thesymptoms of this patient improved significantly with corticosteroids alone due to early detection. This is the first case report of neurotoxicity following toripalimab treatment in a patient with pancreatic cancer. Once the n-irAEs have been managed effectively, and unable to affect the tolerance to the subsequent treatment, it can potentially aid the patient to achieve a longer OS.

Here, we report a case of advanced PDAC patient treated with toripalimab-induced encephalopathy, exhibiting stuttering as the first clinical signal, followed by slowness in thought, and confirmed the presence of multiple cerebral white matter demyelinating imaging changes. The symptom remission was achieved upon discontinuation of toripalimab and corticosteroid therapy. Overall, stuttering might serve as an early symptom of neurotoxicity, which can provide optimal guidance for recognizing this early occult ICI-related n-irAEs that is easily overlooked currently in the clinical practice.

## Data Availability

The original contributions presented in the study are included in the article. Further inquiries can be directed to the corresponding authors.

## Abbreviations

FOLFIRINOX: Fluorouracil, irinotecan, leucovorin and oxaliplatin
ICI: Immune checkpoint inhibitor
irAEs: Immune-related adverse events
MRI: Magnetic resonance imaging
n-irAEs: Neurological irAEs
OS: Overall survival
PDAC: Pancreatic ductal adenocarcinoma
PD-1/L1: Programmed cell death protein 1/ligand 1
PFS: Progression-free survival
T-GA: Toripalimab, gemcitabine and nab-paclitaxel
